# Biofouling of leisure boats as a source of metal pollution

**DOI:** 10.1007/s11356-016-7883-7

**Published:** 2016-10-20

**Authors:** Maria Alexandra Bighiu, Ann-Kristin Eriksson-Wiklund, Britta Eklund

**Affiliations:** Department of Environmental Science and Analytical Chemistry, Stockholm University, 10691 Stockholm, Sweden

**Keywords:** Antifouling paint, Biofouling, Copper, Zinc, Boat, Field study

## Abstract

The release of harmful metals from antifouling paints to water bodies is a well-known problem. In this study, we measured both the amount of biofouling growth on leisure boats during one season as well as the concentration of metals accumulated by the biofouling matrix. Furthermore, the efficiency of antifouling paints and mechanical boat cleaning as well as the effect of hull colour on biofouling were evaluated. Unlike paint residues, biofouling waste has never been regarded as a source of metal contamination and has previously been neglected in the scientific literature. Our results revealed that the biofouling waste contained very high concentrations of metals, up to 28,000 mg copper/kg dw and 171,000 mg zinc/kg dw, which exceeds the guidance values for least sensitive land use in Sweden by factors of 140 and 340, respectively. This observation is important because the contaminated biofouling waste is commonly disposed of in boatyard soils at the end of each season, thus increasing the levels of metal pollution. Moreover, there was no significant difference in the amount of biofouling if the boats were coated with copper or zinc containing paints or no paint at all, indicating that biocide paints might not be necessary in low-salinity areas such as the Stockholm archipelago. For boats that were not painted at all during the season, those washed on boat washers (mechanically) had on average half of the amount of biofouling compared to boats that were not cleaned mechanically. The results of the study indicate the importance of proper management of biofouling waste as well as the use of more environmentally friendly removal methods for biofouling such as boat washers.

**Electronic supplementary material:**

The online version of this article (doi:10.1007/s11356-016-7883-7) contains supplementary material, which is available to authorized users.

## Introduction

Biofouling is defined as the ‘undesirable accumulation of biological material on the surfaces of submerged structures’ (Arai et al. 2009). In the case of ships, biofouling causes increased fuel consumption and may contribute to the spread of invasive species (Dürr and Thomason 2010). The most common measure of biofouling prevention consists of the use of antifouling (AF) paints (European Environment Agency 2013). Owing to the large diversity of fouling organisms (more than 4000 species including bacteria, algae, barnacles, molluscs, etc.) (Dürr and Thomason 2010), it is difficult to find an antifouling formula that can reduce target species without harming non-target species. This issue is particularly important for leisure boats (i.e. boats <25 m), as these vessels sail mainly in shallow coastal areas where many aquatic organisms reproduce and where the water exchange is low. Moreover, leisure boats spend most of their time in ports, thus causing higher levels of local pollution owing to the release of antifouling substances (Finnish Chemicals Agency 2011).

Currently, the most widely used type of AF paint for leisure boats is a ‘self-polishing copolymer’ that acts by slowly releasing biocides into the water (Buskens et al. 2013). These paints contain copper as the primary active ingredient, and in some cases, organic ‘booster’ biocides such as Irgarol 1051®, Sea Nine 211®, zinc pyrithione, dichlofluanid and zineb, among other compounds (European Environment Agency 2013), to increase their efficacy, especially against Cu-resistant algae such as *Ulva* spp., *Achnanthes* spp. or *Ectocarpus* spp. (Voulvoulis et al. 2002). Studies have shown that some of these compounds can persist in the environment, harm non-target species and possibly enter the food chain (Antizar-Ladislao 2008; Sousa et al. 2014). Toxic effects of copper-based AF paint leachates have been noted for several non-target aquatic species such as the macroalgae *Ceramium tenuicorne* (growth inhibition) or the crustacean *Nitocra spinipes* (mortality and effects on larval development ratio) (Karlsson and Eklund 2004; Karlsson et al. 2006; Karlsson et al. 2010).

The use of AF paints in the EU is regulated by the EU Biocidal Products Regulation No. 528/2012. In Sweden, regulation of AF paints currently includes different specifications for the west and east coasts. Thus, because of higher salinity and higher fouling pressure, paints with higher copper content and leakage rate are allowed on the west coast (e.g. up to 35 % copper), whereas on the east coast, only those paints with a maximum copper content of 8.5 % and a lower leakage rate can be used. In the northern part of the Baltic Sea (the Bothnian Bay) and in freshwaters, no copper is allowed. Organic booster biocides are not allowed on leisure boats in Sweden (KEMI-Swedish Chemicals Agency 2011), and therefore, our study is not focused on AF agents containing these compounds. Zinc-based paints have also been used quite extensively in Sweden as well as hard paints such as epoxy and other so-called biocide-free paints (e.g. Lago racing II, Neptune formula).

Other non-coating methods are also available for use on boats in the Baltic Sea; the most widely used method is the mechanical cleaning of boat hulls in boat washers. There are approximately 16 of these devices in small boat harbours around the Baltic Sea, and they work by in-water brushing of the boat hulls. Collection of biofouling material and paint waste occurs on plastic sheets (tarpaulin) and the material is then further treated as hazardous waste. This method, considered less harmful compared to the use of AF paints, is encouraged in several municipalities in Sweden, but its use remains infrequent. A survey carried out in 2010 estimated the total number of leisure boats in Sweden to be approximately 507,000 (excluding small boats such as kayaks or motorboats <10 hk), out of which approximately half used AF paints (Swedish Transport Agency 2010). This phenomenon presents a problem because the Baltic Sea is categorized as a ‘particularly sensitive sea area’ and hosts key species (e.g. *Fucus vesiculosus*, *Mytilus edulis* and *Gadus morhua*) (KEMI-Swedish Chemicals Agency 2012) that are potentially endangered by the presence of AF biocides (Finnish Chemicals Agency 2011).

Antifouling paints do not only affect the quality of the aquatic environment but can also contaminate sediment and soil in the areas adjacent to harbours (Eklund et al. 2008; Eklund et al. 2010). This contamination is due mainly to hull cleaning operations such as scraping and sandpapering as well as to the storage of boats on land during winter (Turner 2010; Turner 2013; Eklund et al. 2014; Eklund and Eklund 2014). Very high concentrations of metals (Pb, Cd, Hg, Cu, Zn), as well as organic contaminants (TBT, PAHs and PCBs), accumulate on the top layer of soil in boatyards as a result of boat maintenance activities (Eklund and Eklund 2014). In addition to the environmental risk, these contaminants also pose a health hazard, as the workers in charge of removing paint from boat hulls have a potential body exposure loading of up to 33 mg copper h^−1^ during sand blasting (Links et al. 2007). Moreover, despite the 25-year ban of tributyltin (TBT) paints (Directive 89/677/EEC), TBT is still being released from old paint layers during high water pressure cleaning or sandpapering of leisure boat hulls. For example, a study by Eklund et al. (2008) noted concentrations of TBT up to 2000 μg/kg dw in the surface sediment in a harbour in the Baltic Sea. In Australia, total TBT concentrations ranging between 220 and 8759 μg/kg were found in the sediment of a commercial marina (Burton et al. 2005). Today, TBT paints are also prohibited for ships since the adoption of the AFS convention (International Convention on the Control of Harmful Antifouling Systems on Ships), which has been enforced since September 2008 (International Maritime Organization 2001). There are many more studies that show evidence of the environmental problems caused by the use of antifouling paints (Antizar-Ladislao 2008; Matthiessen 2013; Sousa et al. 2014).

In this study, we focused on the importance of biofouling waste as a source of metal pollution to boatyard soils, an issue which (to our knowledge) has never been addressed before. The main questions this paper seeks to answer are as follows: (i) what amounts of copper, zinc and tin are released from antifouling paints into boatyards via the biofouling material? (ii) How much biofouling is accumulated on leisure boats sailing in brackish water? (iii) How efficient are different antifouling paints and boat washers in keeping boat hulls free from biofouling? (iv) Is there an effect of hull colour on the amount of biofouling?

## Materials and methods

The data were obtained through field observations, including questionnaires, and complemented by a field experiment. The boats investigated in this study were located in three main harbours in the Stockholm area where the water salinity varies between 2.5 and 5 ppt (59° 19′ 46.8″ N 18° 09′ 13.3″ E, 59° 21′ 18.4″ N 18° 03′ 03.3″ E and 59° 22′ 26.5″ N 18° 03′ 22.8″ E).

### Biofouling sampling

Sampling of biofouling from boat hulls was performed immediately after the boats were taken out of the water for dry-docking (i.e. October, 2012). Only 2 out of the 104 boaters approached refused to participate in the study. Thus, biomass was collected from a total of 102 boats (30 motorboats and 72 sailing boats) representing two different boat clubs in Stockholm, Sweden. The lengths of the boats ranged from 4 to 12 m, with an average of 7.9 ± 1.5 m. For practical reasons, the boats were selected in the order in which they were taken out of the water.

**Fig. 1:**
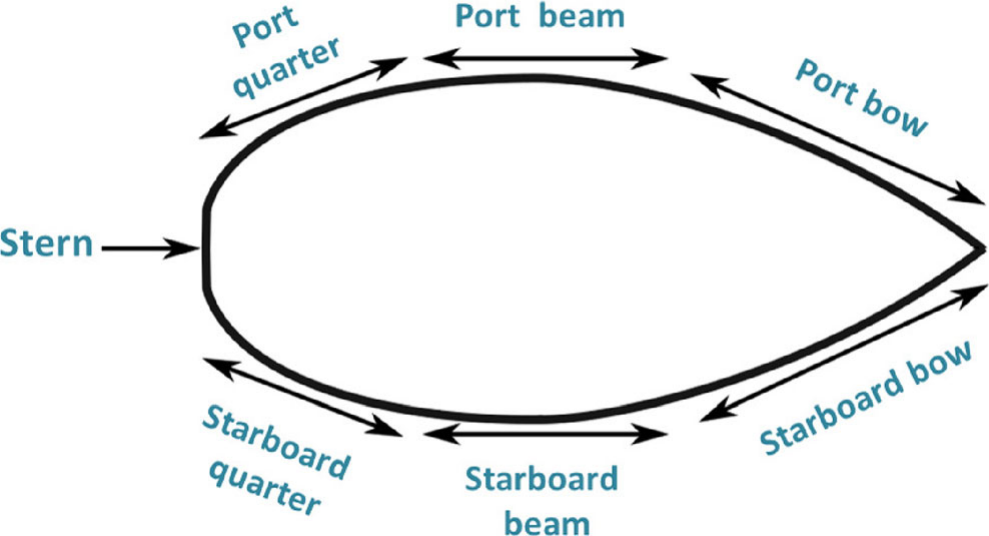
Areas of each boat from which biofouling was sampled

To obtain a representative estimation of the biofouling coverage, seven samples were taken per boat from different areas of the hull, which included the starboard (bow, beam, quarter), port (bow, beam, quarter) and stern (Fig. 1). Biofouling covering areas between 2 and 8 dm^2^ was removed a few centimetres below the waterline using rubber scrapers. The scraped material was then placed into preweighted bags, transported in coolers and frozen at −20 °C once it reached the laboratory. The biofouling material was then dried in the oven at 60 °C for 2–3 days, after which the dry weight was measured. No species identification was performed for the field study.

To correlate the amount of biofouling on boats to different methods for biofouling removal or prevention, questionnaires were used during the field sampling for gathering information directly from the boat owners. Some of the most relevant information included (1) the type and amount of paint used during the current season, (2) if a boat washer was used, (3) boat age and (4) the main harbour in which the boat was placed as well as the general sailing area. The full questionnaire can be found in the Annex.

### Metal analysis on boat hulls and in the biofouling material

A subset of 30 boats was randomly chosen for metal measurement directly on the hulls with a new X-ray fluorescence (XRF) application described by Ytreberg et al. (2015). A handheld XRF-analyser (Delta 50, Olympus, Innovx, USA) was used to quantify the area concentrations of tin, copper and zinc in antifouling paints applied on boat hulls. Tin was measured as an indication of the presence of organic tin in antifouling paints. The method is fast (30 s) and non-destructive, and the limits of detection are 13.3, 23 and 2.9 μg/cm^2^ for Cu, Zn and Sn, respectively. For easier comparisons with the metal levels in the biofouling material, the area concentrations were converted from μg/cm^2^ to μg/g, using the instrument’s collimator area of 0.305 cm^2^ and the average weight of a dry paint layer of 0.042 g.

Biofouling samples collected from the same 30 boats mentioned above were chemically analysed for Cu, Zn and Sn according to the Swedish Standard SS 02 81 50–1. Because these samples were also weighed for quantifying biomass, they could not be rinsed prior to analysis owing to the risk of losing material and thus creating a bias in the biomass values. The dried biofouling was autoclaved in 20 % nitric acid at 125 °C for 30 min and diluted with MilliQ water prior to analysis using inductively coupled plasma mass spectrometry (ICP-MS, Thermo Scientific X-series 2). The internal standards used were ^45^Sc (20 μg/L), ^103^Rh (10 μg/L) and ^187^Re (10 μg/L). The isotopes used were ^65^Cu, ^66^Zn and ^120^Sn. Our own metal solutions served as references, which were as follows (averages and standard deviations): 24.67 ± 1.33, 25.32 ± 0.97 and 5.061 ± 0.258 μg/L for Zn, Cu and Sn, respectively. No correction for the blanks was needed.

### Colour experiment

Initial observations revealed a significant interaction between the type of antifouling paint and the colour of the boat hull (data not shown), and these observations led to the design of a ‘colour experiment’ in the following season. Thus, in an attempt to study the effect of the surface colour independently from the effect of the active substances in the paints, an experiment was conducted for 21 days at the Stockholm University field station at Askö (100 km south of Stockholm). This area is located on an island and is uncontaminated by any industrial outlets or sources of antifouling paints. Plastic used for food packaging was chosen as substratum, assuming that it does not leach toxic substances. Several precautions were taken for controlling the cleanness of the plastic material. Firstly, the metal content of the plastic was measured with the same XRF application as the boat hulls. The results revealed very small concentrations of Zn (average 8.3 ± 7.08 ppm) and Cu (87.55 ± 15.42 ppm). These concentrations correspond to 0.02 and 1.2 % of the average concentrations of Zn and Cu found in the biofouling (see the “Metals in biofouling material vs boat hulls” section) and were thus considered negligible. The detailed results for each colour are found in Table 4 in the Annex. Secondly, to remove organic residues, the plastic panels were washed with alkaline RBS T105 (Borghgraef S.A., Belgium) and subsequently rinsed for 24 h in Milli-Q water. Plastic panels with an average area of 69.4 ± 15.2 cm^2^ were attached on PVC bars at a distance of approximately 2 cm from each other. The bars were then exposed in situ, at a water depth of 1.5 m. The panels were fixed in a vertical position with their front side facing north-east (NE) or south-east (SE) and the back side facing towards the floating platform they were attached to (i.e. the back side was shaded). The most common colours of boat hulls were used for this exposure, i.e. black, white, blue and red (*N* = 35 for each colour) as well as transparent panels (*N* = 22) that served as substratum controls. All panels had similar surface texture. Light and temperature were measured on each side of the platform (NE and SE) throughout the experiment using loggers (HOBO®, Onset) placed at the same depth as the panels.

After the settlement period of the barnacles (i.e. mid-August), the panels were taken out of the water and the main groups of macrofoulers were identified and counted on both sides of the panels and the biomass from the front side of each panel was dried and quantified.

### Data analysis

The data were statistically analysed using JMP software v. 11 (SAS, USA). The boats were divided into categories based on the main ingredient of each paint, i.e. *copper*, *zinc*, *other* (biocide-free paints such as epoxy and other similar hard paints) and *no paint* (boats that have not been painted during the season). It is important to note that paints categorized as ‘copper’ may also contain some zinc, whereas the paints from the ‘zinc’ category contain only zinc. Univariate analysis was performed through ANOVAs (for data normally distributed, followed by Tukey HSD) and Kruskal-Wallis tests (followed by Steel-Dwass comparisons for data not normally distributed) for investigating differences in biofouling due to different colours or paints, among other factors. Non-parametric regression (Spearman’s ρ) was used for analysing the relationship between metal concentrations on boat hulls and in the biofouling material. The level of significance was set at 0.05 for all tests.

## Results and discussion

### Biofouling

The boats included in the study were covered almost exclusively by soft biofouling, with algae representing most of the biomass. Only five of the investigated boats had barnacles. Salt from the ambient water was considered to have a negligible effect on the sample weight, as the salinity in this area is very low, i.e. 2.5–5 ppt.

The degree of biofouling did not differ among boats belonging to the different harbours (*p* = 0.71, *df* = 3, F ratio = 0.47), and thus the data were pooled for the statistical analysis. The average amount of biofouling was 0.073 g dry weight/dm^2^, which resulted in a total of 146 g of dry biomass for a regular leisure boat with a hull area of 20 m^2^. The distribution of biofouling across different regions of the boat hulls is shown in Table 1.

**Table 1:** Amount of biomass on different regions of the boat hull (g/dm^2^)

	Port			Stern	Starboard		
	Bow	Beam	Quarter		Bow	Beam	Quarter
Average	0.061	0.057	0.076	0.104	0.076	0.058	0.077
St dev	0.087	0.075	0.101	0.087	0.145	0.07	0.084

The amount of biofouling on the sterns (average 0.104 g/dm^2^) was significantly higher than the amount on the ports (average 0.064 g/dm^2^, *p* < 0.0001) and the starboards (average 0.071 g/dm^2^, *p* < 0.0001). The beam side of the hull had the lowest amount of coverage, followed by the bow side, whereas the quarter side and stern had the highest biofouling (the boat sides are illustrated in Fig. 1). There was no difference in biofouling between the starboard and the port. Visible paint residues were observed on only five of the biomass samples. The amount of biofouling was found to increase proportionally with boat age (*p* = 0.02, *df* = 1, F ratio = 5.57), but there was no clear mechanism for this relationship.

### Metals in biofouling material vs boat hulls

Copper was detected on 85 % of the boats, with 73 % of them having levels above 2000 μg/g, and zinc was found on 85 % of the boats, with most values (81 %) higher than 8000 μg/g. For comparison, one layer (i.e. 46 μm dry thickness) of a typical brackish water AF paint contains approximately 8000 μg/g copper and 14,000 μg/g zinc (Ytreberg et al. 2016). Sn was detected on 89 % of the boats, though only 23 % were higher than 500 μg/g, indicating the possible remnants of TBT paints in deeper layers. The metal measurements on boat hulls (Table 2) exceeded the values found in paint flakes by Rees et al. (2014) by a factor of between 2.5 and 90 for copper and 1.7 and 383 for zinc (based on median values). The results of our study also exceeded those by others (Turner et al. 2015) for Cu and Zn, whereas Sn concentrations were lower by a factor of 8.5. The high levels of metals detected in the current study may represent both an environmental risk as well as a health hazard for people performing the paint scraping (Links et al. 2007).

**Table 2:** Concentrations of copper (Cu), zinc (Zn) and tin (Sn) measured in the biofouling material with ICP-MS and in the antifouling paints, directly on boat hulls using XRF; SL and LSL are the Swedish guideline values for *sensitive* and *least sensitive land use*, respectively

	Biofouling (μg/g)			Boat hulls (μg/g)		
	Cu	Zn	Sn	Cu	Zn	Sn
median	3471	6253	10	4608	17,646	75
mean	4686	21,650	18	7122	29,568	651
St dev	5506	41,092	21	12,207	31,024	1242
SL	80	250	NA			
LSL	200	500	NA			

To our knowledge, this is the first peer-reviewed study that shows accumulation of metals in biofouling (represented mainly by algae here). The chemical analysis of the dry biofouling revealed metal concentrations ranging between 57.8 and 27,586.1 μg/g for copper, 224 and 171,445 μg/g for zinc and 0.6 and 88.6 μg/g for tin. It is important to note that none of these samples contained any visible paint residues. Descriptive statistics for the metal concentrations are found in Table 2, along with the Swedish guideline values for land use. As the concentrations of Sn were not particularly elevated, the discussion is focused on Cu and Zn. Paradas and Amado Filho (2007) sampled two species of *Ulva* from marinas in Brazil and found copper ranging between 5.4 and 152.32 μg/g and zinc between 14.97 and 174.37 μg/g, which is considerably less than those in our study. Johnston et al. (2011) also measured metal concentrations in the kelp *Saccharina latissima* in marinas and found levels up to 6 and 60 μg/g for Cu and Zn, respectively. One explanation for this large difference could be because in our study, the algae were collected from the boat hulls, as opposed to the seafloor ﻿by Johnston et al. (2011).

**Fig. 2:**
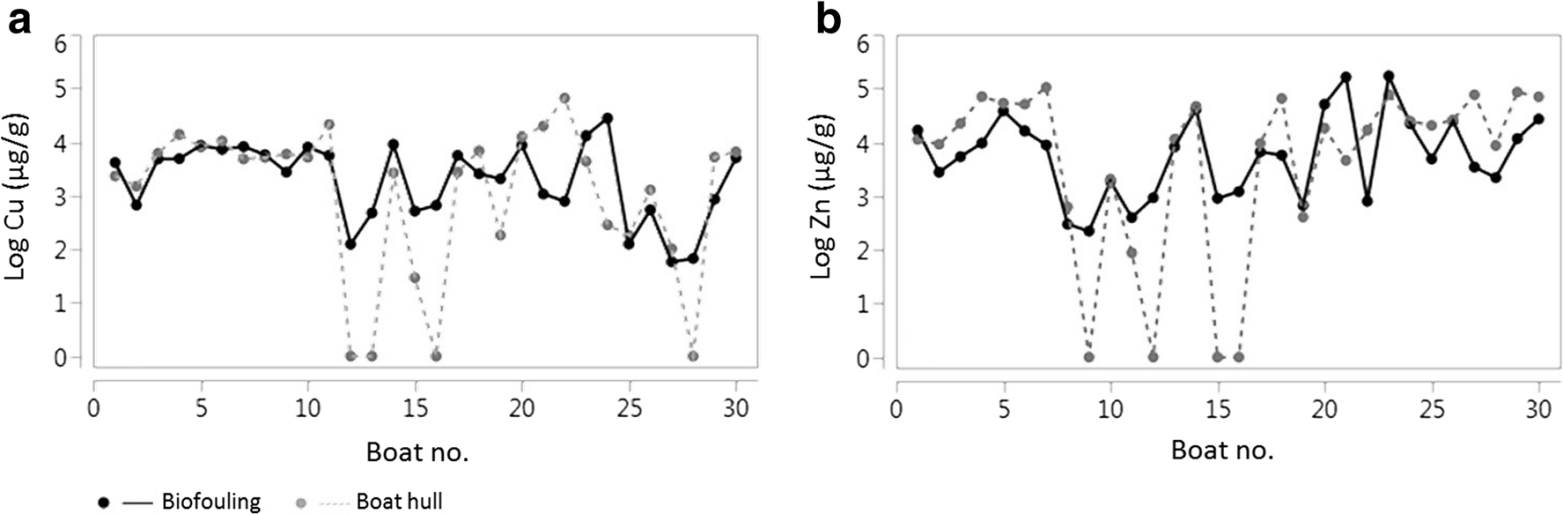
**a, b** Relationship between the concentrations of metals in the biofouling material and on the boat hulls

The concentrations of metals found in the fouling algae in this study are much higher than the background levels in sea lettuce reported by Campanella et al. (2001), i.e. 12.9 μg/g for copper and 50 μg/g for zinc. This result indicates that the algae (which were the main fouling organisms in our study) are able to concentrate copper up to 2100 times and zinc up to 3400 times, presumably both from the antifouling paints used on the leisure boats as well as directly from the harbour water. This indication means that for a regular boat (hull area of 20 m^2^), the average amount of these metals in the biofouling material is 0.7 g copper and 3.17 g zinc. Thus, for a regular boat club with 200 boats, on average, 137 g copper and 633 g zinc end up on the soil every season because of biofouling removal only. This result leads to the exceedance of the Swedish guideline values for least sensitive land use (LSL) by a factor of 23 for Cu and 43 for Zn. Therefore, this heavily contaminated biofouling waste needs to be properly collected and treated. Furthermore, when other hull maintenance activities such as sandpapering or scraping of paints are performed, the amounts of metals ending up on the soil are much higher. For example, studies have shown that the surface soil in boatyards can contain amounts of copper up to 7700 mg/kg dw and zinc up to 5000 mg/kg dw, exceeding the Swedish LSL guidelines by a factor of 10–100 (Eklund and Eklund 2014). There are several indications that the uptake of metals by the fouling organisms occurred mainly from the antifouling paints leaching from boats. Firstly, the amounts of metals in dried biofouling were significantly correlated to the amounts of metals on boats: Spearman ρ was 0.51 for Cu and 0.65 for Zn (*p* = 0.0042 and 0.0001 for Cu and Zn, respectively). Figure 2 shows that the concentrations of metals in the biofouling material reflect those measured on the boat hulls with XRF quite well. On average, the copper amount on the boat hulls was 1.5 times higher than in the biomass and zinc was 1.4 times higher on the boat hulls than in the biomass. Tin (Sn) was 36 times higher on the boat hulls than in the biomass, indicating low uptake by the fouling organisms. Since there was no correlation between the concentration of Sn in biofouling and on the boat hulls, it is likely that Sn is found in a deeper (TBT) paint layer and it is thus not directly available for uptake by fouling organisms. However, the occurrence of tin (as TBT) in underlying paint layers can pose a health risk for people coming into contact with paint flakes or dust during boat maintenance activities such as paint scraping.

**Fig. 3:**
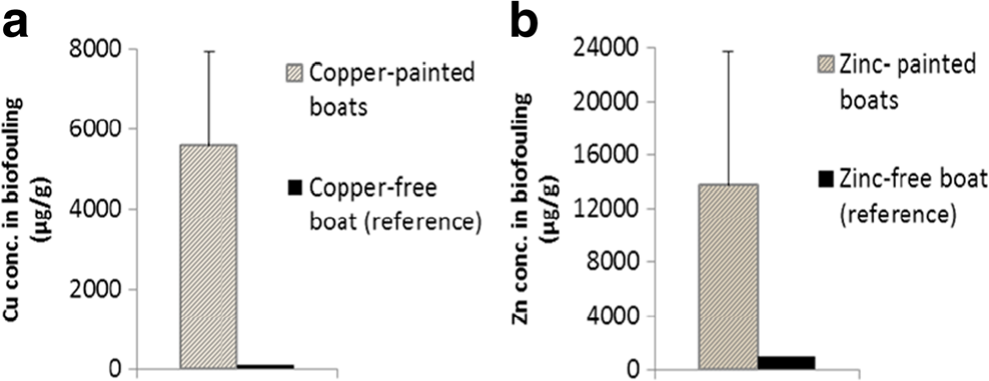
**a, b** Average concentrations of copper and zinc in biofouling sampled from coated vs non-coated boats; *error bars* show standard deviations

Secondly, the uptake of metals from water can be evaluated by comparing the concentrations in biofouling sampled from a reference boat (i.e. a 10-year-old boat which has never been painted) to those sampled from boats coated with copper or zinc-containing paints. Figure 3 shows the high difference in uptake of metals from water vs antifouling paints, implying that the boat hulls are the main source of metals for the fouling organisms. Moreover, the levels of metals in the biofouling material show a positive (though not statistically significant) correlation with the volumes of antifouling paint used on the respective boats (data not shown).

Since copper is taken up in such high amounts by the fouling organisms, its bioavailability is decreased and, therefore, taxa that are generally not copper tolerant can still attach on top of these ‘foundation species’ (Piola et al. 2009). This phenomenon might imply that, despite the constant input of copper paints into the aquatic environment, the problem of the transport of invasive species on fouled vessels might not be solved. In addition to this hypothesis, as shown in the “Antifouling paints” section, the use of copper paints does not make a significant difference in the degree of biofouling on leisure boats sailing in brackish water. These facts suggest that copper paints are currently used to a larger extent than what is actually necessary in a low-salinity environment.

### Antifouling paints

The questionnaires revealed that out of the 102 boats investigated in this study (response 95.1 %), 40 were coated with copper paints, 27 with zinc paints, 7 with other types of paint (e.g. epoxy) and 15 were not painted during the studied season. Thus, approximately 84 % of the boats were coated with some type of AF paint, and this proportion is in accordance with the general situation in the Swedish Baltic Sea, where a survey showed that 80 % of the boat owners use AF paints (Wester 2009). The investigated boats are likely representative of the area studied because they were not selected by any particular criteria but were chosen in the random order in which they were taken out of the water.

A total of 166 L of antifouling paints were used for these boats, with copper and zinc paints representing the highest amounts (56 and 34 %, respectively, of the total volume). Furthermore, 21 % of the boat owners did not know what type of paint their boats were coated with. One of the reasons for their unawareness was that some of the people bought their boats in that season and had no knowledge of the previous coatings used. However, half of them have owned their boats for more than 10 years, so the lack of knowledge is rather surprising. Thus, it seems that more awareness and responsibility should be employed by boaters to have control of the extent of use of antifouling paints in the Baltic Sea. Moreover, 14 % of the boat owners admitted to have used paints that are not allowed in the area studied and this is a problem well-known from other studies too (Swedish Transport Agency 2010).

One of the arguments commonly used by boaters in support of the use of antifouling paints is that they wish to combat the settlement of barnacles. However, in this study, barnacles were present on five boats only, regardless of which maintenance method was used. This observation indicates that fouling due to barnacles in particular might not be a very large problem on the Northern part of the Baltic Sea, where the water salinity is low. Nevertheless, seasonal variations in larval availability may also have contributed to the reduced settlement of barnacles in the investigated period.

**Fig. 4:**
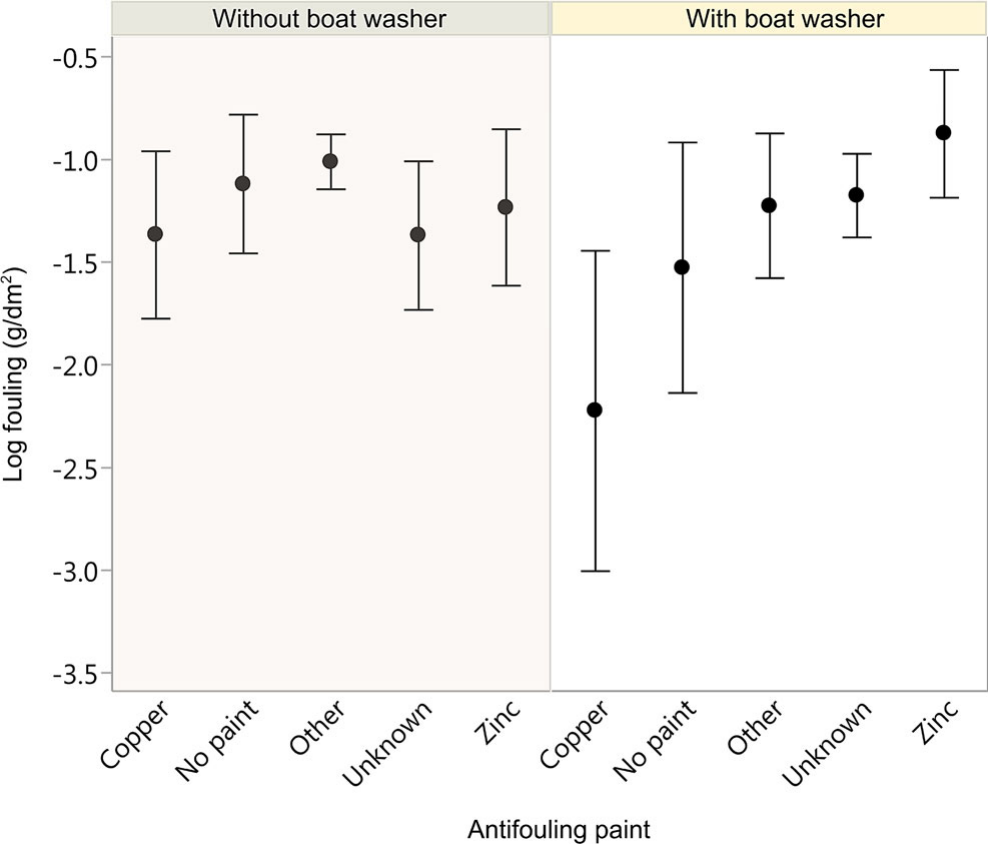
Dry fouling (log scale) in relation to different AF paints, without and with the use of boat washers; means ± sd

The univariate analysis showed that there is no clear evidence of the effect of antifouling paints on the observed amount of biofouling (*p* = 0.11, *df* = 4, F ratio = 1.94). This result is unexpected because it is generally accepted that copper paints are more efficient than are zinc or other biocide-free paints. In addition, copper paints were most efficient only when used together with boat washers (*p* = 0.0004, *df* = 1, F ratio = 15.12, Fig. 4). Ideally, only boats not coated with biocide paints, at least during the respective sailing season, should be washed on boat washers to avoid the enhanced leaching of paints in the water.

With regard to the copper-based paints, no difference in biofouling was observed (*p* = 0.45, *df* = 2, F ratio = 0.81) among boats coated with different copper concentrations (e.g. <8.5, 13 and 17 %). Overall, no difference in biofouling was found between boats that used any type of paint and boats that were not painted at all during the season (*p* = 0.53, *df* = 1, F ratio = 0.4). Thus, it is possible that the paints continue to be active for a longer time than generally thought, and therefore, it might not be necessary to recoat the boat hulls every season, as it is commonly performed at present.

It is well-known that copper is toxic in concentrations higher than those physiologically required. Its toxicity is mainly due to its ability to denature proteins, leading to abnormalities in development and respiratory, productivity, feeding and growth rates (Lewis and Cave 1982). Moreover, copper was shown to affect the olfactory sense of crustaceans, thus impairing their ability to avoid predators and find shelter and food (Simbeya et al. 2012). Zinc is known for causing lethal gill damage in fish (dos Santos et al. 2012), decreases in haemoglobin concentration (Ciji and Bijoy Nandan 2014) and pathological changes in Ca^2+^ and Na^+^ homeostasis in fish (Loro et al. 2014). Therefore, it is important to minimize the input of these metals into the aquatic environment, especially in situations where their use is not really needed.

### Boat washing

Approximately 22 % of the boats in this study were cleaned on boat washers (the rate of response to this question was 100 %). These boats had slightly lower biofouling (e.g. on average 7 % less biofouling) than those not cleaned mechanically, though the difference is not statistically significant (Fig. 4). Half of these mechanically washed boats were also coated with AF paints; consequently, in some cases, it is difficult to conclude which treatment was responsible for the observed amount of biofouling. It is also important to note the limitation of not knowing when the boats were cleaned mechanically during the season. Nevertheless, in the case of those boats that were not painted at all during the season, those washed mechanically had, on average, half of the amount of biofouling of those that were not cleaned on boat washers (i.e. average biofouling was 0.06 vs 0.11 g/dm^2^, not significant).

Currently, to our knowledge, there are no studies on in-water hull cleaning methods such as boat washers published in any international, peer-reviewed journals. There are, however, a few studies describing the efficiency of other hull cleaning methods with very different modes of action compared to the ones used in the Nordic countries. One study carried out in Florida (Tribou and Swain 2010) showed that light cleaning (‘grooming’) of boat hulls can enhance the performance of AF paints, but that method is very different from boat washing (brushing) which is meant to be an alternative rather than a complement to AF paints. Another study carried out in Australia (Floerl et al. 2005) gave opposite results, as it showed that manual scrubbing of boats actually enhances the recruitment of some fouling organisms. A survey conducted in 2007 on 231 boats revealed that 71 % of the boat owners in Trosa municipality in Sweden used boat washers and 74 % of them were satisfied with the result (Johansson et al. 2007). Thus, further investigations should be performed to have a complete assessment of the efficiency of this method, as the present study is the only one quantifying the reduction in biofouling through mechanical cleaning on boat washers.

### Colour of boat hulls

The main macrofoulers identified on the panels included the green algae *Ulva* sp. and *Cladophora* sp., the mussel *Cerastoderma glaucum*, the barnacle *Balanus improvisus* and ostracods. Clear species-specific colour preferences were observed in this experiment.

**Fig. 5:**
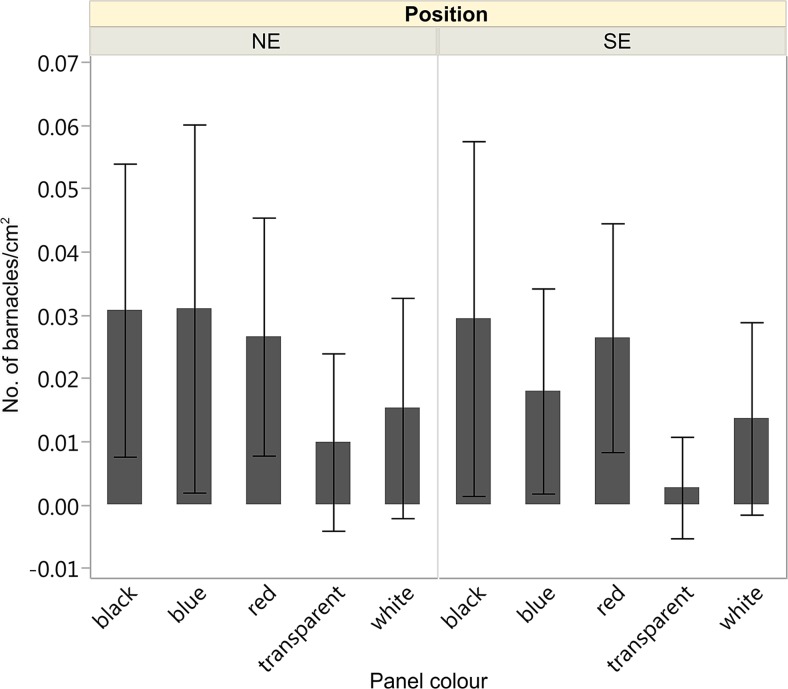
Density of barnacles (*Balanus improvisus*) settled on panels of different colours, facing NE and SE; means ± sd

There was a strong effect of the substratum colour on the settlement of barnacles (*p* < 0.0001, *df* = 4, chi sq. = 26.95), which preferred darker panels (i.e. black, blue and red), while white and transparent panels had the least barnacles (Fig. 5). These results are in accordance with previous studies (Hurley 1973; Satheesh and Wesley 2010; Dobretsov et al. 2013) in which barnacles settled most abundantly on dark surfaces (blue, red, black). Significantly lower densities of *Balanus improvisus* were found on panels on which the algae *Ulva* and *Cladophora* were present (*p* = 0.036 and 0.049, respectively), indicating possible competition for space between these species.

**Fig. 6:**
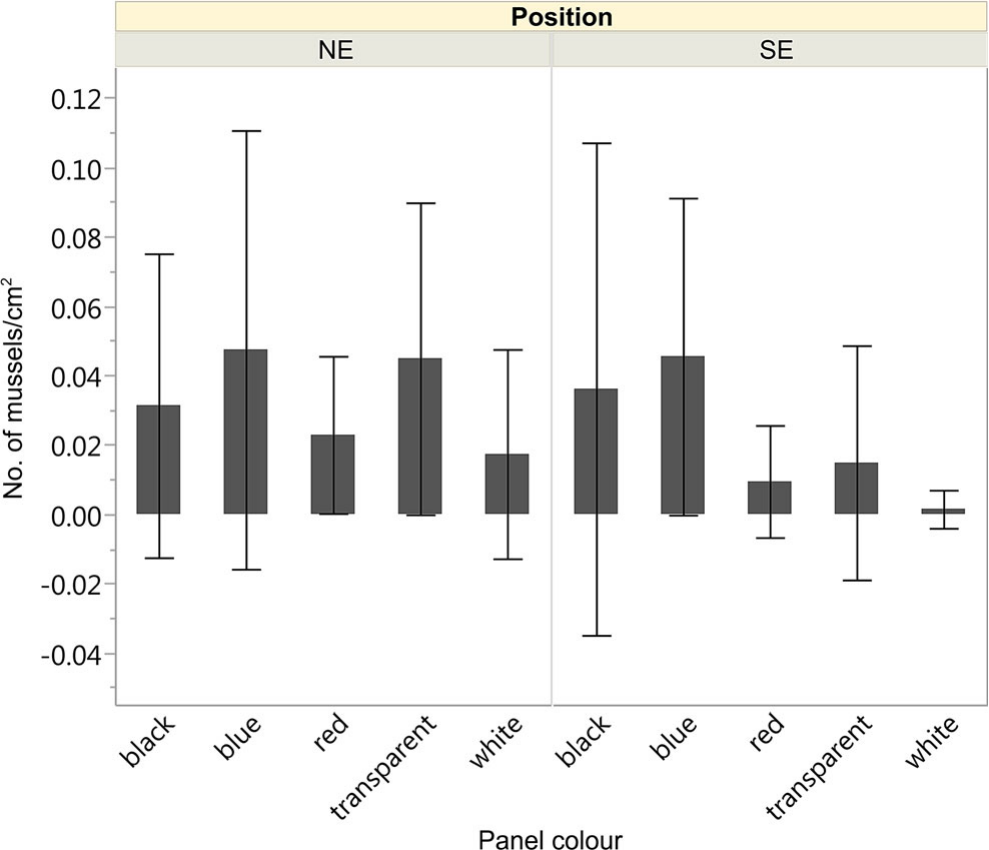
Density of mussels (*Cerastoderma glaucum*) settled on panels of different colours, facing NE and SE; means ± sd

The attachment of the mussel *Cerastoderma glaucum* was also different among colours (*p* = 0.0023, *df* = 4, chi sq. = 16.58, Fig. 6), with white panels having significantly less mussels than do blue or black ones (the comparisons between all pairs are found in Table 3 in the Annex). The mussels were also more abundant on the panels facing NE (*p* = 0.024, *df* = 1, chi sq. = 5.12). The settlement of ostracods was significantly higher on panels oriented towards SE, regardless of colour (*p* = 0.012, *df* = 1, chi sq. = 6.28). For both positions, control (transparent) panels had significantly higher densities of ostracods than black, blue or white panels (Table 2 in the Annex).

Both algae were more frequent on panels oriented towards SE (*p* = 0.027 and *p* < 0.0001 for *Ulva* and *Cladophora*, respectively; data not shown). This result occurred probably because the average light intensity during the experiment was higher on the SE side than on the NE side (i.e. 3236.51 vs 1567.42 lx). The average water temperature during the experiment was 22 °C.

The amount of biofouling in the panel experiment did not follow the same pattern among the colours as observed for the boat hulls. In the panel experiment, the highest biofouling was on red panels, followed by black, blue and white, while in the boat study blue hulls had almost twice more fouling than the rest, followed by red, white and black hulls (data not shown). One of the main reasons for these differences is that, in the field study, several substances leaking from the antifouling paints or other contaminants from the harbour waters could have affected the fouling organisms, while the panel testing was performed with non-toxic materials in a clean area. Other possible explanations include the different exposure times (ca. 5 months for the boats and 3 weeks for the panels), the geographical and seasonal differences in the fouling communities as well as other physical factors that we did not investigate, such as water current, texture and orientation of the surface or differences due to chemical cues created by biofilms (Dobretsov et al. 2013).

Nonetheless, the present study shows that surface colour is an important factor influencing the attachment of fouling organisms. Similar observations were made by Satheesh and Wesley (2010) in India, where the highest biofouling during the summer season was on blue and red panels, compared to white or yellow ones. Swain et al. (2006) also showed the importance of surface colour in short-term testing of antifouling paints, with black panels being the most heavily fouled. Our results suggest that coating the boat hulls with lighter colours may help in minimizing biofouling to a certain degree. For example, white boat hulls had approximately 9 % less biofouling than did the black ones, 6 % less than did the blue ones, and 17 % less biofouling compared to red hulls. However, it is important to keep in mind that the actual formulation of the active substance in the paint (e.g. copper thiocyanate vs copper oxide) can lead to very different levels of biofouling, in which case the effect of surface colour might not be observed.

The levels of biofouling on the boats were also analysed in relation to the different areas where the sailing took place (see the questionnaire in the Annex). Since the boats in the present study were stationary approximately 91 % of the time (i.e. average number of sailing days in the season was 13.8 ± 11.89), no difference in biofouling was detected due to sailing area in particular.

## Conclusions

The integrated approach of our study made it possible to relate the levels of metals in the biofouling material to the boat hull maintenance methods, assess the efficiency of these methods and examine the role of other factors that may influence biofouling. Thus, owing to the very high concentrations of metals accumulated in the biofouling material, we emphasize the need for improving the current management of hull cleaning activities by proper collection and treatment of the biofouling waste.

Regarding the efficiency of antifouling paints, there was no significant difference in biofouling between the different categories of paint, meaning that copper-based paints are not necessarily more efficient than others are in preventing biofouling on boats sailing in brackish water. Therefore, we conclude that copper-based antifouling paints are currently used to a higher extent than needed in low-salinity water. Thus, future studies should be performed on more environmentally friendly methods such as mechanical cleaning of boat hulls, which would reduce both the environmental risk and the health hazard for workers within boat maintenance facilities.

## Electronic supplementary material

ESM 1 (PDF 551 kb)
