# Supplementary material for: Biofouling of leisure boats as a source of metal pollution
Source: Environ Sci Pollut Res Int. 2016 Oct 20;24(1):997–1006. doi: 10.1007/s11356-016-7883-7 (PMC5219026; doi:10.1007/s11356-016-7883-7)
Supplement: Supplementary file 1 — (PDF 551 kb) [file 11356_2016_7883_MOESM1_ESM.pdf]

Biofouling of leisure boats as a source of metal pollution

Environmental Science and Pollution Research

Maria Alexandra Bighiu[1*], Ann-Kristin Eriksson Wiklund[1], Britta Eklund[1]

[1]Department of Environmental Science and Analytical Chemistry, Stockholm University, Sweden

[*]corresponding author: maria.bighiu@aces.su.se, Svante Arrhenius väg 8, SE-10691, Stockholm, Sweden, phone +46 8 674 7609

Supplementary information

A. Questionnaire (adapted from Swedish)

Q1. How satisfied are you with the aspect of the boat hull as regards fouling? Rank from 1-5 (not satisfied-very satisfied).

Rate of response: 91.2 %

Average grade: 3.85 ± 1.15

Q2. What type of antifouling paint did you use in the last season?

Rate of response: 95.1 %

- Copper paints: 41.2 %
- Zinc paints: 27.8 %
- No paint: 15.5 %
- Other (biocide free paints including epoxy) 7.2 %
- Unknown 8.2 %

Q3. How many litres of antifouling paint did you use in the last season?

Rate of response: 90.2 %

- Copper: 2.4 ± 1.8 L
- Zn 2.2 ± 1.2 L
- Other: 0.82 ± 1.09 L

Q4. Did you use any kind of boat washer in the last season (e.g. manual or industrial)?

Rate of response: 100%

- Yes: 22 %
- No: 78 %

Q5. How many labour hours did you put in the boat maintenance in the last season?

Rate of response: 93.1 %

- Average 9.3 ± 17.7 h

Q6. How much money did you invest in boat maintenance activities?

Rate of response: 88.2 %

- Average 936.7 ± 1210 Swedish Crowns

Q7. Which was the main area in which you have sailed in the last season?

Rate of response: 98 %

- Only inner Stockholm archipelago: 38 %
- Only middle Stockholm archipelago: 12 %
- Only outer Stockholm archipelago: 11 %
- Inner, middle, outer and/or other areas nearby Stockholm: 39 %

Q8. Which was the home harbour for the boat?

Rate of response: 96 %

- Brunnsviken: 24.5 %
- Ekhagen: 29.6 %
- Hundudden: 36.7 %
- Other: 9.2 %

Q9. How many days did you sail in the last season?

Rate of response: 97 %

- Average 13.8 ± 11.8 days

Q10. How was the boat positioned in the harbour (according to the stern)?

Rate of response: 92.1 %

- E: 24.5 %
- W: 26.6 %
- N: 10.6 %
- NE and NW: 14.9 %
- S: 13.8 %
- SE and SW: 9.6 %

Q11. How old is your boat?

Rate of response: 97 %

- Average 35.44 ± 14.3 years

In addition, we made observations of hull colour and type of boat (motor or sailing boat).

B. Colour panels experiment

Table 3. Multiple comparisons of the densities of the three groups of macrofoulers between the different substratum colours tested. Only the significant differences are shown, obtained using the nonparametric Steel-Dwass method (α=0.05)

Position	*Balanus improvisus*		Ostracods		*Cerastoderma glaucum*	
	colors	p value	colors	p value	colors	p value
South-East	transparent-red	0.0125	transparent-black	0.0004	white-black	0.048
	transparent-black	0.0154	white-black	0.0006	white-blue	0.0058
			black-red	<0.0001		
			blue-red	0.0464		
			blue-black	0.0121		
North-East	transparent-red	0.0316	black-red	<0.0001		
	transparent-black	0.0284	black-transparent	0.0001		
			black-white	0.0027		
			blue-red	0.0335		
			blue-transparent	0.0468		

Table 4. Average metal concentrations in the plastic panels, measured by XRF (N=5 for each colour); nd is not detected

Panel colour	Cu		Zn		Sn
	average	st dev	average	st dev	average
black	90.76	4.74	1.79	1.57	nd
blue	109.31	2.92	11.09	1.19	nd
red	71.46	2.02	17.84	0.72	nd
white	78.69	8.26	2.43	3.75	nd
